# Drivers of Subjective Well-Being Under Different Economic Scenarios

**DOI:** 10.3389/fpsyg.2021.696184

**Published:** 2021-08-11

**Authors:** Rubén Arrondo, Ana Cárcaba, Eduardo González

**Affiliations:** University of Oviedo, Oviedo, Spain

**Keywords:** subjective well-being, life satisfaction, quality of life, Spain, economic downturn

## Abstract

This paper explores the evolution of the driving forces that shape individual subjective well-being (SWB) in Spain from 2013 to 2018. Several socio-demographic, material conditions and quality of life (QoL) variables are considered as potential drivers of SWB. The data come from a large survey carried in two different time periods. The first one (2013) is characterized by a negative economic scenario as a result of the global financial crisis of 2008. The second one (2018) is characterized by fast economic recovery. Our results suggest that the material conditions variables, especially unemployment, have a much deeper impact on SWB during economic downturns than during economic recovery periods. Social connections and health status are determinant factors behind SWB, especially if the economy is working well. Our results also point to changes in gender effects. While women were happier than men in 2013, this effect disappears in 2018. Paradoxically, this suggest an increase in female expectations about their own lives during this period.

## Introduction

The search for happiness is an old aspiration of human beings. More than 2300 years ago, the ancient Greeks were already discussing seriously about the nature of happiness and the paths to achieve it. Aristotle illustrated the importance of happiness and well-being for human development: “Happiness is the meaning and purpose of life, the general and final goal of human existence.” Recent times have witnessed the renaissance of happiness centrality in policy. Even the Davos’ 2019 World Economic Forum dedicated a special section to happiness and well-being. In one of the sessions, New Zealand Prime Minister Jacinda Ardern expressed with clarity the central role of the topic: “We need to address the societal well-being of our nation, not just the economic well-being.”

The limitations of traditional income measures as indicators of social progress, has led to a search for new concepts that represent more accurately the goals of society. There are many international initiatives aimed at measuring happiness in order to quantify social progress and encourage governments to use this information to implement policies that can have an impact on people’s lives. Among the most influential, we may highlight the OECD’s Better Life Index, the World Database of Happiness or the Happy Planet Index, among others. One of the most influential is the Better Life Index (BLI), which compares well-being across countries, focusing on 11 dimensions of life that the OECD qualify as critical. These include quality of life (QoL) and material conditions variables that condition life.

The modern academic interest in the discipline of happiness has evolved around the generic notion of subjective well-being (SWB). The literature often uses the terms SWB, QoL, Life Satisfaction and Happiness interchangeably, which reflects certain overlap between these concepts at both theoretical and empirical levels. However, notable differences exist, mainly with respect to SWB and QoL. Camfield and Skevington (2008) provide a deep discussion of the differences and commonalities between these concepts. To avoid confusion, in this paper we make a fundamental distinction between SWB and QoL that is common within the literature on well-being. Our starting point is Diener (1984) definition of SWB as a way of defining the scope of psychology that seeks to encompass people’s self-assessments of their life satisfaction, which includes both their cognitive judgments and their affective reactions (Diener et al., 1997). An individual’s level of SWB relates both to objective conditions of life (QoL) and to individual factors that affect the self-perception of life experiences. More precisely, the potential level of SWB is a response to genetic and environmental elements, dimensions that interact continuously (Okbay et al., 2016). While an important part of potential SWB is imprinted in our genes (Lykken and Tellegen, 1996), research shows that the environment is at least as much important. The environmental features that drive individual SWB are a combination of socio-demographic, material and quality of life factors. Empirically, there is cross-section evidence that relates these elements to SWB (Arrondo et al., 2020).

Less attention has been paid to studying the time evolution of SWB in a population. External shocks (such as economic crises or pandemic conditions) may have notable effects not only on the levels of SWB but also on the elements that configure SWB itself. The 2008 economic crisis had a profound economic impact on the financial situation of large parts of the population. It also brought emotional suffering from facing sudden and unexpected changes, which may have an effect over mental models of happiness. For instance, unemployment is a factor that affects SWB very negatively. However, the way this variable affects SWB in a scenario of deep economic crisis may be much more severe than the way it does in a growing economic scenario. It is the same variable, but the expectations and emotional implications it brings may be completely different (Deaton, 2012). On the other hand, a period of economic recovery may as well have an impact by restoring to new emotional balances that may affect the individual’s perception of well-being.

The aim of this work is to examine which factors have been the main drivers of individual SWB in Spain over the period 2013–2018 and to explore the changes in the importance of the different driving forces over time. In doing so, we follow the definition of well-being elaborated by the OECD in the BLI project (Durand, 2015). The BLI proposal classifies the dimensions in two blocks: material conditions and QoL variables. The main variables reflecting QoL are health status, education and skills, social connections, environmental quality, work-life balance, civic engagement and governance, personal security and social security. In turn, material conditions refer to income and wealth, employment, and housing. Our empirical analysis is inspired by Boarini et al. (2012), who related the SWB variable to the other 10 dimensions of the BLI. Therefore, QoL and material conditions variables are taken as the driving forces behind the general levels of individual SWB. On the other hand, we will also investigate how socio-demographic variables such as age, gender, marital status or region relate to the perception of SWB. It is a fundamental goal of this paper to analyze how the socioeconomic transition from a period characterized by a severe economic downturn (2013) to one of economic recovery (2018) affected the general satisfaction with life of the population.

The first set of data available for this study comes from year 2013 in which Spain was still suffering the severe impact of an economic recession that hit the world since 2008. The prospects of economic recovery seemed remote then. Global macroeconomic factors (unemployment, inflation) will have an impact on the way SWB is obtained at the individual level, with QoL variables, especially personal relationships and health, being moderating factors (Gonza and Burger, 2017). In periods of economic recession, important changes occur in magnitudes that are very relevant for people’s lives, especially in those associated with material living conditions: income and wealth, housing and unemployment. Unemployment was the main problem for 81.6% of the Spanish population in 2013, according to the studies of the Spanish Centro de Investigaciones Sociológicas. Losing a job is a crucial event that causes a huge drop in people’s life satisfaction (Clark et al., 2008; Hahn et al., 2015). The adverse effects of unemployment on SWB are probably the most agreed relationship in the SWB literature (O’Connor, 2020)^1^. Our view is that this effect is not independent of the overall level of unemployment. This is, being unemployed in a crisis scenario is much worse for SWB than in a recovery scenario. Guardiola and Guillen-Royo (2015) have verified that during crisis contexts with high levels of unemployment, higher education and employment status are strong determinants of SWB. In order to explore these possibilities, we count on a second set of survey data that refers to period 2018, when the Spanish economy was showing a continued rebound, with high growth figures and a reduction of unemployment rates from 26% to 15% (OECD Economic Surveys: Spain 2018).

## What Drives SWB?

Academic analysis of SWB has focused on knowing the reasons why people feel satisfied or dissatisfied with their lives. While individual perceptions of life may have a genetic base, are subjective and unique to each person, research points to certain measurable factors that have substantial effects on SWB (Sirgy, 2019). Wilson (1967) seminal work identified a series of factors that contribute to SWB, attributing happiness to youth, health, good education, good salary, extroversion, optimism, carefreeness, religiosity, self-esteem, work morale and modest aspirations. The first three variables (health, education and income) have long been considered as the cornerstones of human well-being. The Human Development Index (HDI), created in 1990 by the United Nations, focused precisely on these three dimensions in order to make social progress comparisons among countries. The objective of the HDI was to shift the focus of policy from economic growth to social welfare. However, the specific manner in which each of these three elements contributes to SWB is a matter of debate in the literature.

Most studies find income as having a favorable impact on the level of satisfaction with life of individuals (Sacks et al., 2010), although the exact relationship between both variables remains unclear. Diener and Biswas-Diener (2002) review of the literature noted that the impact of income on SWB is positive if it means avoiding poverty or living in a developed country. In contrast, the domestic effect of income in developed countries is more limited. Individuals’ aspirations moderate the effect of income and, in turn, past income feeds into aspiration levels. Consequently, Dolan et al. (2008) state that it is unlikely that additional income, for people in reasonably high-income levels, will lead to substantial increases in SWBs. This income paradox was noted earlier by Easterlin (1974, 1995) who found that individuals with greater wealth in a given society are happier, although happiness does not increase as much as income does. This reminds of the ubiquitous economic law of decreasing returns. When income is sufficiently large, other factors may have a greater influence on happiness, so that the marginal effect of income becomes insignificant.

Additionally, comparisons introduce another important moderating factor for income. Both comparisons with other people belonging to the same social group (social comparisons) and comparisons with oneself in the past (habituation). Di Tella et al. (2010) showed strong evidence of the small long-term effect of a change in income on SWB. Relative income, rather than income itself, would be the actual driver of happiness (Clark et al., 2008). Thus, it is not surprising that the impact of income on happiness is greater in developing countries than in developed ones.

At the same time, the link between education and happiness is equally vague. While education could increase SWB because it prepares individuals to better manage and deal with environmental situations, it could also raise aspiration levels, thus decreasing happiness (Graham, 2012). Some studies present evidence of a positive and significant effect of education (Blanchflower and Oswald, 2004). In contrast, other studies have shown that the effect of education is small after examining and controlling for the impact of education on income and health (Helliwell, 2008). As for health, the empirical evidence is less unequivocal. Physical and psychological health are key determinants of SWB (Dolan et al., 2008). Disability has a negative and lasting effect on the life satisfaction of individuals with disability (Lucas, 2007). And healthy habits are also found to be strongly associated to life satisfaction (Grant et al., 2009). Additionally, there are very important gender inequalities related to health status that have an influence well-being (Merino-Llorente and Somarriba, 2020).

The three variables involved in HDI are to some extent interrelated. People with education and studies tend to obtain better employment and better health habits. In turn, people with greater wealth invest much more in education and personal health. Likewise, a healthier person is more likely to find better jobs and a higher level of education. For these reasons, the individual and separate effect of each of these variables on SWB becomes difficult to identify. In addition, important indirect effects may exist among these variables. For example, some studies have found significant indirect effects of education on SWBs due to their effect on health (Gerdtham and Johannesson, 2001; Bukenya et al., 2003).

Although income, education, and health are the main drivers of SWB, the list of variables that to a greater or lesser extent have an effect on SWB is much wider. The OECD’s Better Life Index provides a very comprehensive description that distinguishes a first set of material conditions variables from a second set of QoL variables. According to this proposal, the material conditions are represented by income and wealth, jobs and earning, and housing. In turn, the list of quality of life variables includes health status, work-life balance, education and skills, social connections, civic participation and governance, environmental quality, and personal safety.

Therefore, some important dimensions of life can be added to the well-known HDI triad of income, education and health. In terms of material conditions, jobs and earnings (a dimension related to unemployment) and housing add important facets to income and wealth. Clark and Oswald (1994) and Oswald (1997) observed that individuals attribute a subjective cost to unemployment in terms of loss of SWBs well above that which would correspond to the respective loss of income. Furthermore, unemployed individuals no longer return to previous SWB levels when they get a new job (Lucas et al., 2004). Thus, it is apparently unemployment, and not working, that diminishes life satisfaction. In general, individuals who do not work but are not unemployed (retirees, students, etc.) do not report lower levels of SWB (Blanchflower and Oswald, 2004). Finally, a number of studies have found a positive relationship between the quality of the housing and SWB (Zumbro, 2014; Herbers and Mulder, 2017; Zhang and Zhang, 2019), which also exerts indirect positive effects on health status (Kemeni, 2001).

With respect to QoL variables, the BLI adds work-life balance, social connections, civic engagement and governance, environmental quality and personal security to health and education. As noted above, the employed are happier and more satisfied with their lives than the unemployed, although the number of hours worked also influences the SWB. Meier and Stutzer (2006) found an inverse U-shaped relationship between hours worked and life satisfaction. This means that full-time work increases SWB relative to part-time work, but only up to the point where the number of hours worked becomes excessive. This means that there must be a correct balance between work and time available for personal activities. In the same vein, time spent travelling to work is an element that decreases SWB because it reduces the time available for leisure or productive activities (Stutzer and Frey, 2005). Furthermore, time spent caring for others has a negative impact on life satisfaction, and this, too, explains the reduced effect of having children on reported SWBs (Dolan et al., 2008).

Civic engagement, including involvement and participation within the community, has consistently shown a beneficial effect on SWB (Helliwell, 2003; Meier and Stutzer, 2006). With respect to governance and participation, Rodríguez-Pose and Maslauskaite (2012) observed that good governance has a positive influence on happiness. Similarly, transparency and good governance in public administrations also positively affects individual well-being in the field of municipal studies (Frey and Stutzer, 2000; Cárcaba et al., 2017).

Scholars have long noted the beneficial contribution of close social connections to SWB (Cohen and Wills, 1985; Helliwell and Putnam, 2004; Puntscher et al., 2015; Liu et al., 2017). Socializing with family and friends has a very positive influence on SWB (Kahneman and Krueger, 2006; Lelkes, 2006). A common driver of SWB is marital status. According to Argyle and Furnham (1983), people who are married are happier because marriage brings a very strong social bond, with both material and emotional support. These effects also seem to include stable, unmarried partners (Brown, 2000). Likewise, trust and social support from others are strong drivers of well-being (Helliwell and Wang, 2011). Consequently, social connections and cohabitation with a partner have a major positive influence on SWB.

Although the evidence in the literature is not very extensive, the quality of the environment has also been positively associated with SWB (Welsch, 2002, 2006; Ferrer-i-Carbonell and Gowdy, 2007) and happiness (MacKerron, 2012). Noise pollution also has an impact on life satisfaction (Weinhold, 2008). Access to green spaces in cities helps mitigating stress and promoting mental well-being (Liu et al., 2021). Studies show a positive association between green spaces and SWB (Ambrey and Fleming, 2014; Ma et al., 2018; Mavoa et al., 2019). However, the relationship between natural outdoor environments close to the city and SWB may be moderated by several factors such as sociodemographic and socioeconomic variables, specific characteristics of the natural environments, climatic conditions, etc. (Avolio et al., 2015). At the same time, evidence suggests that, controlling for income differences, residing in an unsafe area negatively impacts SWB (Lelkes, 2006; Ferrer-i-Carbonell and Gowdy, 2007; Balestra and Sultan, 2013).

In addition to material conditions and QoL variables, demographic characteristics may also be important determinants of SWB. With regards to gender, an early meta-analysis conducted by Haring et al. (1984) concluded that men had higher life satisfaction than women. In contrast, a later review of specialized literature found men as having lower levels of life satisfaction than women (Wood et al., 1989). Again, Pinquart and Sörensen (2001) concluded in their meta-analysis that men had slightly higher levels of life satisfaction. Finally, a recent review of studies by Batz-Barbarich et al. (2018) found no significant gender differences in life satisfaction after controlling for different types of biases. This result is counterintuitive, since social and institutional structures generate all sorts of gender differences and inequalities that could also affect SWB. The evidence regarding aging is more robust. Some research finds a positive effect (Argyle, 2013), while other researchers find a U-shaped type of relationship (Ferrer-i-Carbonell and Gowdy, 2007; Blanchflower and Oswald, 2008; Sujarwoto et al., 2018).

**FIGURE 1:**
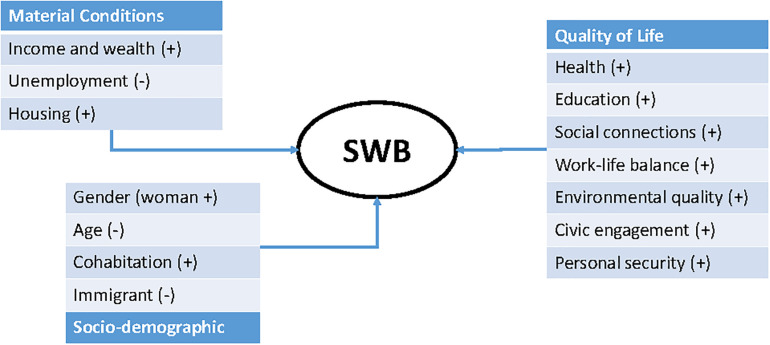
Main drivers of SWB.

Figure 1 summarizes the different drivers of SWB discussed above. All the material conditions and all the QoL variables, except unemployment, have an expected positive influence on SWB. The expected effects of the socio-demographic variables are more specific. We expect women, cohabiting partners and nationals to have higher average levels of SWB, which will decrease with aging. The effect of aging could also be U-shaped.

## Dynamics of SWB

The time evolution of SWB depends to a great extent on the evolution of its driving forces. Therefore, improvements in material conditions and quality of life variables should be accompanied by an increase in SWB. Looking at the individual level, some variables can change very quickly, while others cannot. For example, an individual may lose the job and become unemployed from one day to another. Health status may also deteriorate on time or improve quickly after receiving treatment from an illness. Other variables operate only in one direction. Education is the most notable, since you can only go forward. Within these limits, the expectation is that the evolution in the level of SWB may relate to the evolution in the levels of its driving forces.

However, the manner in which the different driving forces affect the level of SWB may also evolve on time. External events may change the way individuals perceive the effects of their personal situation concerning those forces. The financial crisis that shook the world in 2008 provides an opportunity to check these variations in the weights attached to the different dimensions that make a good or a bad living. A large portion of the population personally suffered the effects of the crisis during the years after 2008. Some lost their jobs, other lost their houses (houses they could no longer afford) or found no finance for their housing prospects. Under these circumstances, it is reasonable to expect that income, housing and unemployment, i.e., the material conditions variables, would play a central role in the way the individual perceives SWB.

But the well-known Easterlin paradox (Easterlin, 1974) points to an imbalance in the way material conditions and SWB would evolve on time. Namely, as material conditions improve above some certain level, SWB stagnates or at least diminishing returns appear. Therefore, we should expect a reduction in the relative importance of material conditions variables after recovery from an economic crisis. Therefore, our expectation is that during periods of economic downturn, material conditions variables would gain importance as drivers of individual SWB. In contrast, during periods or economic recovery, QoL variables would increase relative importance.

Additionally, social connections may have a moderating effect on SWB in periods of economic downturn, through the instrumental support of friends and family when searching for job opportunities or obtaining financial support. Moreover, social connections bring emotional support and a sense of belonging that helps maintaining the levels of SWB (Helliwell and Putnam, 2004). Social connections may help mitigate psychological overloads by improving the ability of the individual to deal with situations that involve stressful events (Ross and Jang, 2000; Kawachi and Berkman, 2001). In short, social connections exert a positive influence in alleviating anxiety and fostering positive thoughts and feelings. There is a virtuous circle between good social relations and present and future individual well-being.

## Data

The data comes from the annual survey on living conditions in Spain, elaborated by the Spanish National Institute of Statistics (INE) for the years 2013 and 2018. We took these years, because they are the only ones that contain a supplementary module with information on well-being. Indeed, INE introduced the well-being module in the 2013 survey and is including it again every 5 years. This circumstance provides a unique opportunity to compare the evolution of SWB over time on a large dataset. The target population includes both households and individuals, with a sample size around 13,000 households and 35,000 individuals. These individuals are selected through a two-stage stratified sampling method and then, interviewed in person. Unfortunately, the data does not constitute a panel, since the respondents are different in 2013 and 2018. The reason is that 25% of sample respondents are replaced every year. Therefore, every 4 years, the sample is completely renewed.

The dependent variable is the usual Satisfaction with Life scale, which reports the individual’s level of satisfaction with current life from 0 to 10. As for the drivers of SWB, our data contains variables that can approximate the QoL and material conditions status of the individual surveyed. Concerning the material conditions, these three variables are included:

- Income and wealth: Disposable income of the household (thousand Euros)
- Jobs and earnings: Unemployment (1 unemployed, 0 other situations)^2^
- Housing: Real market value of the dwelling (real or likely rent of the dwelling in relation to the cost of living)

In turn, the next seven variables account for the QoL dimensions:

- Health status: Perception of own health (0--10 scale)^3^
- Work-life balance: Satisfaction with leisure time (0–10 scale)
- Education and skills: Education attainment (0--10 scale)^4^
- Social connections: Satisfaction with personal relations (0–10 scale)
- Civic engagement and governance: Social trust (0–10 scale)
- Environmental quality: Of the house and surroundings, including pollution and noise (0–10 scale)
- Personal security: Perception of delinquency in the zone (0--10 scale)^5^

We also include four socio-demographic variables:

- Age: Expressed in years
- Gender: 0-Man, 1-Woman
- Cohabiting: 0-Single, 1-Married or cohabiting with a stable couple
- Immigrant: 0-Immigrant, 1-Spanish

In summary, we have one measure of SWB, four socio-demographic variables, three indicators of material conditions and seven QoL variables. We established some filters for selecting the final sample for our study. First, we are only interested in the population between 18 and 65 years of age (working age). Second, we imposed the condition that all the variables contain a valid value for each individual. After applying these filters, the final sample size was reduced to 19,026 and 20,175 individuals for the years 2013 and 2018, respectively.

Table 1 displays a description of the data. The sample is almost equally split by gender and the average age for the individuals surveyed is around 43–44. In both periods, the great majority of the individuals interviewed are Spanish nationals (90 and 87%, respectively), with around 60% living with someone. Regarding SWB, the variable satisfaction with life has an average of 6.96 in 2013 (close to the EU-28 average, which was 7.10). As expected from a period of economic recovery, the average of SWB experienced a substantial rise in 2018, reaching a value of 7.46. Regarding the material conditions, the average income and wealth reported for 2013 and 2018 was 36.3 and 39.3, respectively, reflecting again the effects of economic recovery. In contrast, the housing variable remained very stable during both periods. The 2008 downturn hit hard on employment. We note these effects since unemployment dropped from 22% in 2013 to 14% in 2018.

**TABLE 1:** Descriptive statistics.

**Variable**	**Avg. 2013 (SD)**	**Avg. 2018 (SD)**	**Global min**	**Global max**	**Avg. diff. *T*-test**
**N**	19,026	20,175			
**SWB**	6.96 (1.94)	7.46 (1.71)	0	10	26.4***
**Socio-demographic**					
Age	43.00 (13.05)	44.13 (13.21)	18	65	10.1***
Immigrant	0.10 (0.29)	0.13 (0.33)	0	1	9.6***
Gender (female)	0.51 (0.49)	0.51 (0.49)	0	1	0.3
Cohabiting	0.62 (0.48)	0.61 (0.49)	0	1	−0.5
**Material conditions**					
Income and wealth	36.3 (23.4)	39.3 (24.8)	0	325	12.3***
Housing	0.30 (0.17)	0.31 (0.19)	0.01	4.33	7.7***
Unemployment	0.22 (0.41)	0.14 (0.35)	0	1	32.4***
**Quality of life**					
Health status	7.40 (1.94)	7.54 (1.95)	0	10	14.3***
Work-life balance	6.38 (2.36)	6.68 (2.22)	0	10	13.0***
Education and skills	5.87 (2.98)	6.40 (2.99)	0	10	16.2***
Social connections	7.83 (1.65)	8.25 (1.46)	0	10	26.9***
Civic eng. and govern.	6.28 (2.05)	6.69 (2.15)	0	10	19.2***
Environmental quality	8.64 (2.76)	8.65 (2.77)	0	10	0.28
Personal security	8.63 (3.42)	8.95 (3.06)	0	10	9.2***

*Standard deviations in brackets. ^∗∗∗^ Significance level 0.01.*

As for the QoL variables, all of them improved during the period analyzed. Environmental quality, personal security and social connections obtain very large average scores in both periods. On the opposite side, education and skills and civic engagement and governance are the dimensions with lowest averages. These figures evidence weaknesses in the education system and distrust with the political and legal system. Fortunately, we see some steps in the right direction, since education raised from 5.87 to 6.40 during the period considered. It is also noteworthy the improvement in social connections, from 7.83 to 8.25.

**FIGURE 2:**
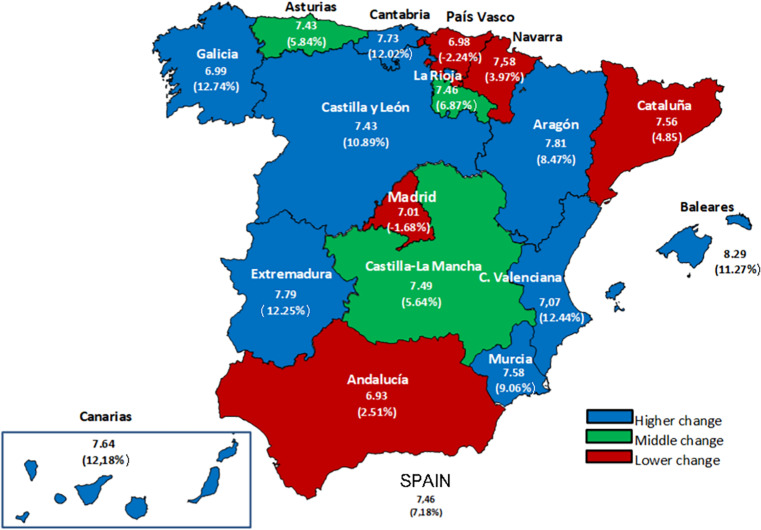
Geographic distribution of SWB by regions in 2018 (change in brackets).

Figure 2 shows the geographic distribution of SWB in the Spanish territory for 2018 and the variation from 2013. The Autonomous Communities (ACs) or regions with the largest averages are Baleares (8.29), Comunidad Valenciana (7.95) and Aragón (7.81). On the opposite side, Andalucía (6.20), País Vasco (6.98), and Galicia (6.99) show the lowest values. Although we appreciate some differences, the geography does not seem to be a determinant factor. The differences are much smaller than the differences that can be found in terms of income, employment or quality of life (Guardiola and Guillen-Royo, 2015; González et al., 2018).

The evolution of SWB during the period under analysis is more interesting than the average values. The largest improvements are observed in Galicia (12.74%), Comunidad Valenciana (12.44%), Extremadura (12.25%), Islas Canarias (12.18%), Cantabria (12.02%), Baleares (11.27%), and Castilla y León (10.89%). On the other hand, we appreciate a slight regression in País Vasco (−2.24%) and Madrid (−1.68%). Interestingly, these last two ACs were the ones with lowest unemployment figures during the crisis. In contrast, the ACs that were hit hard by the crisis, with large unemployment rates, then registered a fast growth in activity and employment. Some of them are tightly associated with the recovery of the tourism sector (Islas Canarias, Baleares and Comunidad Valenciana).

## Results

The empirical model relates SWB with a number of driving forces related to socio-demographic variables, material conditions of living and QoL variables. Table 2 summarizes the results of the regression analysis with the data of 2013 and 2018. Since the survey is structured by household and, in some cases, more than one individual from the household is interviewed, the observations are not independent and identically distributed. In order to estimate consistently, we used a clustered error regression model in which households are the clusters. We follow Helliwell (2008) and Boarini et al. (2012) in treating the dependent variable is treated as numerical in the linear regression model.

**TABLE 2:** Drivers of SWB in 2013 and 2018.

	**Model 1 2013**	**Model 2 2018**	**Beta coeff. 2013**	**Beta coeff. 2018**
Intercept	0.9523 (5.59)***	1.3401 (8.90)***		
**Socio-demographic**				
Age	−0.0183 (−2.83)**	−0.0166 (−3.14)**	−0.238	−0.128
Age^2^	0.0001 (1.10)	0.0001 (1.35)	0.110	0.065
Immigrant	−0.1193 (−2.54)**	−0.0233 (−0.66)	−0.035	−0.004
Gender (female)	0.0856 (4.23)***	0.0181 (1.09)	0.041	0.005
Cohabiting	0.4841 (15.10)***	0.4637 (17.33)***	0.232	0.130
**Material conditions**				
Income and wealth	0.006 (11.18)***	0.004 (10.42)***	0.140	0.057
Unemployment	−0.9217 (−27.29)***	−0.8282 (−23.45)***	−0.377	−0.169
Housing	0.2162 (2.63)**	0.1817 (3.23)**	0.0367	0.020
**Quality of life**				
Health status	0.1921 (24.36)***	0.1955 (27.72)***	0.372	0.220
Work-life balance	0.1173 (17.07)***	0.1486 (23.78)***	0.276	0.192
Education and skills	0.0317 (6.95)***	0.0317 (8.09)***	0.094	0.055
Social connections	0.3761 (36.65)***	0.3615 (35.61)***	0.624	0.308
Civic eng. and govern.	0.0048 (12.52)***	0.0752 (13.07)***	0.009	0,094
Environmental quality	0.0069 (0.87)	0.0055 (1.18)	0.019	0.008
Personal security	0.0177 (3.81)***	0.0071 (1.49)	0.060	0.012
R^2^	0.3710	0.4114		

*^∗∗^ Significance level 0.05, ^∗∗∗^ Significance level 0.01.*

In both periods, age has a negative and significant effect. However, if we focus on the beta values, this variable was more influential in 2013. This result is consistent with the crisis scenario of 2013 as compared with the recovery picture of 2018. Even with material conditions controlled, the uncertainties introduced with the economic downturn had a special effect on the oldest individuals (within the working ages). SWB is not only affected by having an employment or not or having enough income or not. The positive or negative expectations about the evolution of these variables have a psychological impact, which hits specially the most vulnerable groups. Consistent with this, we also observe how the significant negative effect of being an immigrant (during a crisis period; 2013) turns to be insignificant during the recovery period (2018).

For 2013, our results show a significantly higher level of SWB for women than men, once the rest of the relevant variables on material conditions and QoL are controlled. In 2018, however, we find no significant difference across genders. Analyzing the evolution of SWB from a gender perspective is complicated, since contemporary societies are complex and have undergone profound transformations (Migliorini and De Piccoli, 2020). The WHO (2016) has noticed that women have come a long way in terms of equality and in relation to the social, economic and contextual factors that affect their well-being. In a recent study, Matud et al. (2021) found that women scored higher on life purpose and personal growth, a result in the line of our observation for 2013. However, as we mentioned in Section 2, the literature about gender differences in SWB is inconclusive. Our results point to a convergence between genders. On one side, the gender effect (when controlling for inequalities in material conditions and QoL variables) disappears in 2018. And the uncontrolled difference in SWB averages also disappeared in 2018. While in 2013, women scored higher (6.99 vs. 6.94), in 2018 they score exactly the same (7.45 vs. 7.45). This finding points to some advance in equality across genders, that may arise from a convergence in expectations about life, although this possibility should be studied in depth in future research.

The last of the socio-demographic variables refers to cohabitation. As expected, living with a partner has a very positive and strong effect on self-perceived well-being (independently of being married or not). This favorable association between well-being and cohabitation may be especially relevant in times of economic downturn, as suggested by the Beta coefficients.

Turning to the material conditions variables, all of them have a strong significant effect on SWB. The Beta coefficients point to unemployment as the variable with a greatest (negative) impact on SWB in both periods. We can also appreciate from the Beta coefficients that in 2013 this variable was far more critical than it was in 2018. This finding is again consistent with the expected effects of material conditions during crisis vs. recovery periods. Losing a job during a global downturn of the economy brings more uncertainty and psychological suffering than losing the same job in a recovery scenario. The effects on SWB do not come only from the fact of being unemployed, but on the subjective probability of finding a new job in the foreseeable future. Income and wealth would be the second variable in importance and then housing. These results are also in the line of past research, which document the strong negative effects of unemployment even after controlling for income and other material conditions variables.

Finally, all the QoL variables, except environmental quality and personal safety, have positive and significant effects on SWB in both periods. The three variables with the highest explanatory power in both periods are social connections, health status and work-life balance.

In short, our results point to social connections (a QoL variable) as the mayor single factor explaining SWB in both periods. Regarding the material conditions variables, unemployment is the main factor negatively affecting SWB, being the second factor in order of relative importance. This is a well-documented result in literature on SWB. However, we find that in 2018 (during the recovery phase of the economy), unemployment loses importance and drops to the fourth place (after health status and work-life balance). In other words, we find that during the recovery phase (2018), the three main explanatory factors are QoL variables, while in the crisis phase (2013), unemployment is the second driver with a very large beta coefficient.

The models explain approximately 40% of the variance of SWB. This means there is still another 60% that cannot be explained by sociodemographic, material conditions and QoL variables. We should remember that SWB also has genetic components and is shaped by the personal experience of living. Therefore, it cannot be fully explained by measurable and objective variables. But there are some regularities that drive SWB in most cases in similar ways and relate to the variables examined in this paper. Our results indicate that people who live with someone, has sufficient income, a satisfying social life and good health will be much happier than people do not. Moreover, in times of crisis, unemployment has a much more devastating effect on our perception of well-being than in times of recovery.

## Concluding Remarks

SWB is an individual perception of the own experience of living. As such, it reflects life circumstances, emotions and personality traits (Kahneman and Krueger, 2006). It is also predetermined, to some extent, by genetic factors (Røysamb and Nes, 2019). However, there are some social, demographic and economic variables that regularly drive SWB to a large extent. Knowing the precise direction of these effects and the relative importance of each variable is paramount to support policy makers in the pursuit of sustainable welfare states. Our results point to QoL variables and not material conditions as being the main drivers of SWB in two periods that differ greatly in economic terms. While 2013 was a year in which the devastating effects of the 2008 financial global crisis were hitting Spain hard, our second period (2018) was characterized by a fast recovery of the economy.

The goal of this paper was not only to explore the driving factors behind SWB, but to analyze the time evolution of these relationships when the economic environment changes so dramatically. Some important results have emerged from this exercise of comparing crisis vs. recovery periods. The first has to do with the socio-demographic variables. Aging and cohabiting are very important driving factors of SWB. They have been consistently reported in past literature and they also emerge very clearly in this paper. As people grow older, the levels of SWB tend to go down, although the effect tends to reduce with aging itself. In addition, as expected, living with someone is one of the best recipes for a happy existence. In other words, loneliness is a sure route to unhappiness. These results are identical in both periods and, therefore, seem to be quite consistent.

In contrast, the effects of gender on SWB are puzzling. Past literature has been inconclusive about the effect of being a woman or a man on the self-perception of well-being. In this study, we found that women scored significantly higher in 2013, both controlling and not-controlling for material conditions and other drivers of SWB. Interestingly, this effect has completely disappeared in 2018. One possible explanation behind this empirical finding may be related with a rise in female expectations during the last few years. The influence of the feminist movement toward equality may have (indirectly) increased the expectations of women with regards to their own lives, equating them in fact to those of men. This is a plausible hypothesis that should be analyzed in depth in future research with more data. It is also possible that women are increasingly comparing to other women and not to men, which may attenuate estimated gender differences in the subjective perception of well-being (Shields, 2016). In this line, Bond and Allen (2016) propose overcoming the binarism (male/female) in gender studies to develop a more multifaceted understanding of gender differences. To this end, conducting studies that analyze the differences between groups of women and groups of men separately can help to unravel the relative influence of sociocultural and biological factors, as society exposes different groups to different experiences (Migliorini and De Piccoli, 2020). We leave this exploration open for future research.

With respect to the SWB gap of immigrants the results obtained are less puzzling and can be easily attributed to the evolution of the economy. As expected, in a crisis scenario, immigrants show significant lower levels of QoL. Actually, many immigrants returned their home countries during the economic crisis. When the economy recovered in 2018 this effect disappeared and the results show no significant differences between immigrants and Spanish nationals, regarding SWB (all else being equal).

Our results also confirm the determinant relevance of unemployment in driving SWB. The effect of unemployment is larger than the effect of income, a result also largely documented in the literature. The interesting finding in our results is that the negative effect of unemployment is less determinant during the recovery phase of the economy. This is surprising, since the unemployed is equally unemployed regardless of the evolution of the whole economy. However, the finding is consistent with the well-known fact that the negative effect of unemployment on happiness comes from the fact of being unemployed rather than from the effect of the loss of income. If the effect is a psychological one, the expectations of the unemployed may play an important role. And the evolution of the economy will clearly affect those expectations. This is the psychological effect of being unemployed during a crisis period should be much harder than during a recovery phase. The unemployed that perceives the economy growing fast may hold positive expectations about the future. The prospect of finding a new job is more realistic than during an economic downturn.

Finally, the models estimated also evidence the critical importance of QoL variables in driving SWB, especially when the economy is working well, and material conditions variables lose relevance. Social connections emerge as the single most important driver of SWB in both periods (doubling the beta coefficient of unemployment). Health status is the second driver in 2018 (the third one, right behind unemployment, in 2013). This means that all the policies that may preserve the population in good health and with a stimulating social life will have an enormous impact on SWB.

There are several limitations of our paper that we want to highlight, since they can open new opportunities for future research. First, even though our model explains a considerable portion of the variance in individual SWB, it also leaves much unexplained (around 50%). In other words, our results provide evidence that SWB is highly affected by the material conditions and QoL variables, but still a large part of it depends on personal subjective perceptions, feelings and psychological conditions. Future research should try to isolate the effect of the material conditions and QoL variables, by controlling the individual’s psychological traits. One way of doing this would be by collecting new data on the same sample of individuals, therefore creating a panel. Econometric estimation of panel data model, including individual effects, would be able to account for the unobserved heterogeneity arising from personal factors. Then, time variation in material conditions and QoL variables would determine the effects of material conditions and QoL variables on SWB (*ceteris paribus* the individual). It is our purpose to work on the elaboration of this panel of data.

Finally, it would be desirable to pay further attention to the effects of aging on SWB. Our paper reports a small, almost negligible, effect of aging on SWB. However, our analysis is restricted to working-age individuals. It would be convenient to extend this study with an exhaustive and methodical analysis of the particular indicators that influence SWB in the elderly (older than 65), which are not necessarily the same as the ones included in this study. A deeper focus on a sample of older people would be necessary to assess these differences.

## Data Availability Statement

Publicly available datasets were analyzed in this study. This data can be found here: https://www.ine.es/dyngs/INEbase/es/operacion.htm?c=Estadistica_C&cid=1254736176807&menu=resultados&idp=1254735976608#!tabs-1254736194824.

## Author Contributions

RA revised the literature, worked on the estimations, and prepared the figures in the manuscript. AC obtained the data required for the study, did the computations required in order to be ready for the estimations, and helped with the writing of the manuscript. EG coordinated the work, wrote the final version of the manuscript, and helped in estimating the regressions and discussing results. All authors contributed to the article and approved the submitted version.

## Conflict of Interest

The authors declare that the research was conducted in the absence of any commercial or financial relationships that could be construed as a potential conflict of interest.

## Publisher’s Note

All claims expressed in this article are solely those of the authors and do not necessarily represent those of their affiliated organizations, or those of the publisher, the editors and the reviewers. Any product that may be evaluated in this article, or claim that may be made by its manufacturer, is not guaranteed or endorsed by the publisher.

## Footnotes

1 For an additional and in-depth analysis on the negative effects of unemployment on SWB, please see Lucas et al. (2004), Kassenboehmer and Haisken-DeNew (2009), Tay et al. (2015).

2 A value 0 does not mean the individual is unemployed. The individual could be retired, studying, or other personal not-working situations.

3 The survey reports this variable on a 1–5 scale. A scale change was carried in order to homogenize all the variables to a 0–10 scale. In all cases, in the modified scale 10 indicates the best possible value and 0 the worst one.

4 The survey reports this variable on a 0–5 scale (0-no education received; 1-primary; 2-first stage secondary; 3-second stage secondary; 4-post secondary; 5-higher). It was transformed to a 0–10 scale.

5 From 0 high perception to 10 no perception of delinquency.
